# Interactions of transcranial magnetic stimulation with brain oscillations: a narrative review

**DOI:** 10.3389/fnsys.2024.1489949

**Published:** 2024-12-04

**Authors:** Qijun Wang, Anjuan Gong, Zhen Feng, Yang Bai, Ulf Ziemann

**Affiliations:** ^1^Affiliated Rehabilitation Hospital, Jiangxi Medical College, Nanchang University, Nanchang, Jiangxi, China; ^2^Center for Cognition and Brain Disorders, The Affiliated Hospital of Hangzhou Normal University, Hangzhou, China; ^3^Rehabilitation Medicine Clinical Research Center of Jiangxi Province, Nanchang, Jiangxi, China; ^4^Key Laboratory of Jiangxi Provincial Health Commission for DOC Rehabilitation, Nanchang, Jiangxi, China; ^5^Department of Neurology and Stroke, University of Tübingen, Tübingen, Germany; ^6^Hertie Institute for Clinical Brain Research, University of Tübingen, Tübingen, Germany

**Keywords:** transcranial magnetic stimulation, electroencephalography, brain oscillation, TMS-EEG, enhancement

## Abstract

Brain responses to transcranial magnetic stimulation (TMS) can be recorded with electroencephalography (EEG) and comprise TMS-evoked potentials and TMS-induced oscillations. Repetitive TMS may entrain endogenous brain oscillations. In turn, ongoing brain oscillations prior to the TMS pulse can influence the effects of the TMS pulse. These intricate TMS-EEG and EEG-TMS interactions are increasingly attracting the interest of researchers and clinicians. This review surveys the literature of TMS and its interactions with brain oscillations as measured by EEG in health and disease.

## 1 Introduction

Brain oscillations, first described in the 1920s, are rhythmic patterns of neural activity that result from the complex interplay between the intrinsic properties of individual neurons and the network dynamics of interconnected neuronal populations (Bollimunta et al., 2011; Thut et al., 2012). These oscillations are generated through the orchestrated interplay of ion channel kinetics, synaptic connectivity, and neuromodulatory influences, which together produce the characteristic frequency spectra observed in electrophysiological recordings (Bauer et al., 2006). Neurons possess an intrinsic ability to oscillate across a broad spectrum, spanning from 0.05 to 500–600 Hz (Buzsaki and Draguhn, 2004). The brain oscillations are mainly divided into the following categories: delta (0.5–4 Hz), theta (4–8 Hz), alpha (8–14 Hz), beta (14–30 Hz), gamma (30–100 Hz), fast (100–200 Hz), and ultra-fast (200–600 Hz), but fast and ultra-fast oscillations cannot be measured by electroencephalography (EEG), due to the low-pass filtering characteristics of the skull. The oscillatory activity is not merely an expression of neurons' intrinsic capacity but also a result of their dynamic interactions within and across various brain structures, such as the thalamus, cortex, and hippocampus (Buzsáki and Vöröslakos, 2023). These rhythms can be transient or stable, depending on ongoing cognitive processes and the specific neural context (Swann et al., 2011; Myrov et al., 2024). They can be regional, reflecting local processing within a brain area, or network-wide, indicating the integration of activity across distributed brain regions (Okazaki et al., 2021). The stability and nature of these oscillations are influenced by a balance of excitatory and inhibitory interactions, neuromodulatory inputs, and the structural and functional connectivity of the underlying neural circuits (Agnes and Vogels, 2024). Numerous rhythms have been documented, showcasing variations in their frequency, origin, and responsiveness to alterations in sensory stimuli and task requirements (Buzsaki, 2006; Wang, 2010). Oscillatory activity within specific frequency bands has been associated with distinct brain states and functions. Generally, slow wave activity is associated with sleep, while faster oscillations are linked to the awake state. Delta oscillations seem to play a functional role in coordinating brain activity with autonomic functions, influencing motivational processes linked to both rewards and primal defense mechanisms, and facilitating cognitive functions primarily associated with attention and the recognition of motivationally significant stimuli in the surrounding environment (Knyazev, 2012). The most frequent association of theta oscillations is with memory processes (Klimesch, 1999). Alpha oscillations are indicative of memory processes (Klimesch, 1997) and attentional mechanisms (Hanslmayr et al., 2011). Interactions in the beta-band dominate in tasks that strongly involve endogenous top-down processes. That is, beta is specifically associated with endogenously triggered perceptual changes (Okazaki et al., 2008; Iversen et al., 2009). Gamma oscillations are involved in a wide array of processes, encompassing feature integration, stimulus selection, attention, multisensory and sensorimotor integration, movement preparation, memory formation, and even conscious awareness (Engel et al., 1992; Singer and Gray, 1995; Engel et al., 2001; Fries, 2005; Jensen et al., 2007; Knyazev, 2007; Senkowski et al., 2008; Fries, 2009).

Despite the abundance of high-quality correlational data, this has led to the knowledge that brain oscillations underlie diverse sensory and cognitive processes. Altered membrane properties of cortical and subcortical (especially thalamic) neuronal subpopulations, as well as changes in their connectivity patterns, underlie various neurological and psychiatric disorders (Hughes and Crunelli, 2005; Llinás et al., 2007). These changes may result in significant and noticeable changes in their oscillatory properties, which in turn can affect overall brain function. However, establishing a causal role can only be accomplished through the direct manipulation of these oscillatory signals. Transcranial magnetic stimulation (TMS) is a technique that can be used to study the relationship between brain oscillations and function by directly interacting with brain oscillations. The procedure involves administering a short, powerful magnetic pulse to the head via a coil, which temporarily excites or inhibits the stimulated cortical region (Walsh and Cowey, 2000). It generates electrical currents in a specific area beneath the coil that interact with the ongoing neural activity.

**Figure 1:**
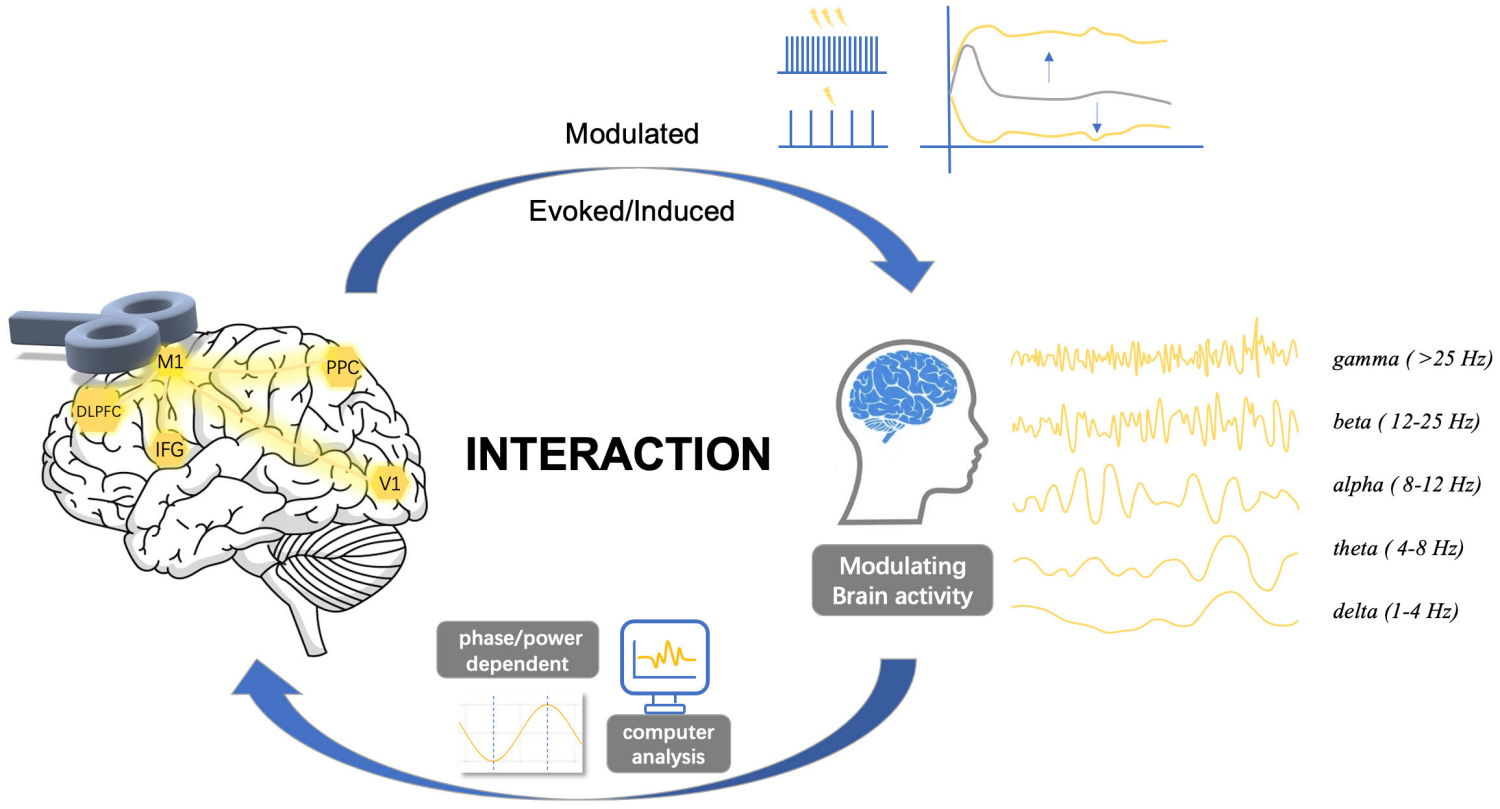
Complex interactions between transcranial magnetic stimulation and brain oscillations. The dynamics of spontaneous neural activity in brain networks suggests a complex interaction between TMS and cortical oscillatory activity. Stimulus site, stimulus intensity, inter-stimulus interval, or pulse configurations may alter cortical states, while the phase and power of cortical oscillations also significantly affect cortical excitability states. This review explores the complex interactions between TMS and brain oscillations, focusing on three key aspects: (1) the effects of TMS on oscillations, (2) the effects of oscillations on TMS, and (3) the bidirectional interactions between them. M1, primary motor cortex; DLPFC, dorsolateral prefrontal cortex; IFG, inferior frontal gyrus; V1, primary visual cortex; PPC, posterior parietal cortex.

The current investigation of brain oscillatory activity using TMS has predominantly relied on three distinct approaches that involve the concurrent utilization of a modulation (i.e., TMS) and a measurement (i.e., EEG). The first approach involves applying repetitive transcranial magnetic stimulation (rTMS) to the brain, where pulses are administered at a specific frequency to a targeted brain region. It modulates the ongoing neuronal oscillations locally and consequently influences brain functions. The second approach involves a combination of synchronized TMS and EEG, where focal single pulses are delivered to induce resonance with the “natural frequency” of the stimulated region. This frequency is determined by the engaged cortico-thalamic modules. It has been proven valuable in studying oscillatory cortical activity, enabling the characterization of the oscillatory patterns of a specific area (Rosanova et al., 2009). Moreover, studies have demonstrated its effectiveness in discriminating between normal and, in clinical populations, abnormal cortical oscillatory patterns (Ferrarelli et al., 2008; Canali et al., 2015; Pellicciari et al., 2016, 2017a). The third approach explores the relationships between on-going oscillations and brain responses to TMS. It also provides a method to investigate the interaction of TMS effects with local oscillatory activity. Thereby, we review here the development and state-of-the-art of the interaction of TMS with brain oscillations from these three aspects (Figure 1).

### 1.1 Search strategy

We searched PubMed for journal articles published in English between Jan 1, 2019, and Aug 1, 2024, for the terms “transcranial magnetic stimulation” or “brain oscillations” or “entrainment” or “brain state-dependent TMS”, and included those papers judged to be most relevant to the focus of this Review. Additionally, we identified and included older papers with ground-breaking findings that led to recent research, using PubMed and by searches of the authors' own files and the reference lists of selected papers.

## 2 Repetitive TMS modulates brain oscillations

Beyond the investigation of oscillatory brain activity using EEG or MEG, repetitive TMS (rTMS) provides a tool to examine the causal relationship between brain oscillations and brain functions by modulating brain oscillations using targeted rTMS frequencies. The field of rTMS is rapidly expanding, exploring the links between intrinsic brain oscillations and specific sensory, motor, and cognitive functions. RTMS holds the potential to engage with or induce local oscillatory activity, serving as a valuable tool for examining the function of ongoing brain rhythms in healthy or diseased brains (Table 1).

**Table 1:** Oscillatory TMS modulates oscillations.

**References**	**Stimulation frequency**	**Measured brain oscillations**	**Subjects**	**Targets**	**Parameters**	**Main findings**
Sokhadze et al. (2009)	0.5 Hz	Gamma (30–80 Hz)	13 Autism 13 HS	Left DLPFC	90% MT 900 pulses	0.5 Hz rTMS over the DLPFC primarily increased gamma (30–80 Hz) responses evoked by non-target stimuli in autism patients.
Schecklmann et al. (2015)	1 Hz	Delta, theta, beta and gamma	20 Tinnitus 20 HS	Bilateral temporal lobe Bilateral prefrontal lobe	60% stimulator output 200 pulses	1 Hz rTMS over the left temporal lobe decreased frontal theta (4–7.5 Hz) and delta (2–3.5 Hz) power, and increased beta (18.5–21 Hz) power. Conversely, right frontal rTMS decreased beta (21.5–30 Hz) and gamma (30.5–44 Hz) power in the right temporal lobe of tinnitus patients.
Strens et al. (2002)	1 Hz	Alpha (7.8–13.7 Hz)	15 HS	Left motor hand area	100% AMT 1,500 pulses	1 Hz rTMS on the M1 caused a significant increase in ipsilateral and interhemispheric alpha (7.8–13.7 Hz) coherence of motor areas.
Tamura et al. (2005)	1 Hz	Beta (15–25 Hz), alpha (7–15 Hz)	12 HS	Left M1	95% RMT 600 pulses	1 Hz rTMS over M1 significantly reduced the movement-related rebound of the 20 Hz oscillation.
Brignani et al. (2008)	1 Hz	Alpha (8–12 Hz), beta (13–30 Hz)	6 HS	Left M1	110% RMT 600 pulses	1 Hz rTMS on the M1 led to the synchronization of the alpha (8–12 Hz) and beta (13–30 Hz) frequency bands in the stimulated region.
Oliviero et al. (2003)	5 Hz	Alpha (10.7–13.6 Hz)	16 HS	Left motor hand area	AMT 50 pulses	5 Hz rTMS over M1 significantly decreased motor-premotor coherence at the upper alpha band (10.7–13.6 Hz).
Fuggetta et al. (2008)	5 Hz	Alpha(10–12 Hz), beta (18–22 Hz)	11 HS	Left M1	80–100%-sham RMT 400 pulses	5 Hz rTMS over M1 induced an increase in alpha (10–12 Hz) and beta (18–22 Hz) power in the frontal, parietal, and central regions.
Dammekens et al. (2014)	10 Hz	Theta and beta	One chronic non-fluent aphasia	Left IFG	80% RMT 2,000 pulses	10 Hz rTMS over the left inferior frontal gyrus increased functional connectivity between the left inferior frontal gyrus (lIFG) and the right inferior frontal gyrus (rIFG) at the theta (4–7.5 Hz) and beta (21.5–30 Hz) frequency bands in chronic non-fluent aphasia.
Xia et al. (2017)	10 Hz	Delta, theta, beta and gamma	18 DoC	Left DLPFC	90% RMT 1,000 pulses	10 Hz rTMS over the DLPFC resulted in a reduction of frontal delta (1–4 Hz), and an increase in frontal and central beta (13–30 Hz) and central gamma (30–45 Hz) in DoC patients.
Veniero et al. (2011)	20 Hz	Alpha	13 HS	Left M1	100% RMT 400 pulses	20 Hz rTMS over M1 led to a dose-dependent enhancement in synchronization within both the alpha (8–12 Hz) and beta (13–30 Hz) bands across central and parietal regions.
Koch et al. (2018)	20 Hz	Beta	14 AD	PC	100% RMT 1,600 pulses	20 Hz rTMS over the left precuneus resulted in an increase in beta (14–30 Hz) power in AD.
Liu et al. (2022)	40 Hz	Gamma	37 AD 41 HS	Bilateral angular gyrus	40% maximal output 2,400 pulses	40 Hz rTMS over the bilateral AG increased gamma-band (30–80 Hz) oscillations in the left posterior temporo-parietal region in patients with AD.

AD, Alzheimer's disease; AG, angular gyrus; AMT, active motor threshold; DLPFC, dorsolateral prefrontal cortex; DoC, disorders of consciousness; HS, healthy subjects; IFG, inferior frontal gyrus; lIFG, left inferior frontal gyrus; M1, primary motor cortex; MT, Motor threshold; PC, precuneus; rIFG, right inferior frontal gyrus; rTMS, repetitive transcranial magnetic stimulation; RMT, resting motor threshold.

Low frequency rTMS, such as 0.5 Hz and 1 Hz, has been shown to have the ability to alter brain oscillations. In most studies the left dorsolateral prefrontal cortex (DLPFC), primary motor cortex (M1), or temporal lobe were stimulated. Low-frequency rTMS typically decreases excitability of the stimulated cortex, but due to the physiological or pathological state of the individual, it can also have opposite effects. For instance, 0.5 Hz rTMS over the DLPFC primarily influenced gamma (30–80 Hz) responses evoked by non-target stimuli in autism patients (Sokhadze et al., 2009). RTMS with 1 Hz pulses on the M1 caused a significant increase in ipsilateral and interhemispheric alpha (7.8–13.7 Hz) coherence of motor areas (Strens et al., 2002). This leads to the synchronization of the alpha (8–12 Hz) and beta (13–30 Hz) frequency bands (Brignani et al., 2008), resulting in a significant reduction of the movement-related rebound in the beta (15–25 Hz) oscillation (Tamura et al., 2005). Additionally, left temporal 1 Hz rTMS decreased frontal theta (4–7.5 Hz) and delta (2–3.5 Hz) and increased beta (18.5–21 Hz) power, whereas right frontal rTMS decreased right temporal beta (21.5–30 Hz) and gamma (30.5–44 Hz) power in tinnitus patients (Schecklmann et al., 2015).

Excitability-increasing high-frequency rTMS could also modify brain oscillations in both healthy individuals and patients. For instance, 5 Hz rTMS over M1 significantly decreased motor-premotor coherence in the upper alpha band (10.7–13.6 Hz) (Oliviero et al., 2003) and induced an increase of alpha (10–12 Hz) and beta (18–22 Hz) power in the frontal, parietal and central regions in healthy individuals (Fuggetta et al., 2008).

Ten Hz rTMS of the left inferior frontal gyrus increased functional connectivity between the left inferior frontal gyrus and the right inferior frontal gyrus in the theta (4–7.5 Hz) and beta (21.5–30 Hz) frequency bands in chronic non-fluent aphasia (Dammekens et al., 2014). Ten Hz rTMS at the left DLPFC resulted in a reduction of frontal delta (1–4 Hz) and an increase in frontal and central beta (13–30 Hz) and central gamma (30–45 Hz) among patients with disorders of consciousness (Xia et al., 2017).

Twenty Hz rTMS of M1 led to a dose-dependent enhancement in synchronization within both the alpha (8–12 Hz) and beta (13–30 Hz) bands across central and parietal regions in healthy subjects (Veniero et al., 2011). Twenty Hz rTMS of the left precuneus resulted in an increase in beta (14–30 Hz) power in patients with Alzheimer's disease (Koch et al., 2018). Additionally, 40-Hz rTMS over the bilateral angular gyrus increased gamma-band (30–80 Hz) oscillations in the left posterior temporo-parietal region in patients with Alzheimer's disease (Liu et al., 2022).

## 3 Single-pulse TMS induced/evoked oscillations

The rhythmic patterns of neural oscillations are believed to play a functional role in local processing and communication between different neuronal systems (Fries, 2005; Thut et al., 2012). Previous research on studying the oscillatory signals at both macro- and micro-scales has led to the emergence of the concept of natural frequencies of the human cortex. It implies that different regions of the cortex inherently exhibit oscillations at varying frequencies (i.e., natural frequencies), which reflect the tuning characteristics of each region (Niedermeyer, 2005). A reliable strategy for determining the cortical natural frequency involves combining TMS with concurrent EEG.

Single-pulse TMS, concurrently with EEG (TMS-EEG) recording, offers a well-suited approach for exploring the natural frequency of specific regions within the brain. Through TMS-EEG, researchers aim to uncover new information about how different areas of the brain are interconnected and integrated. This could ultimately lead to breakthroughs in our understanding of neurological disorders and pave the way for more effective treatments (Table 2).

**Table 2:** Single-pulse TMS induced/evoked oscillations.

**References**	**Measured brain oscillations**	**Subjects**	**Targets**	**Main findings**
Canali et al. (2015)	Beta and gamma	12 MDD 12 SCZ 12 BPD 12 HS	Premotor cortex	The main oscillations of M1 to TMS were significantly reduced in patients with BPD, MDD, and SCZ (11–27 Hz) relative to HS.
Paus et al. (2001)	Beta (15–30 Hz)	7 HS	Left M1	A brief period of synchronized activity in the beta range in the vicinity of M1 was induced by single-pulses TMS.
Van Der Werf and Paus (2006)	Beta	12 HS	Left M1	A transient oscillation in the beta frequency range is induced by single pulses of TMS.
Van Der Werf et al. (2006)	Beta (15–30 Hz)	8 PD	Bilateral M1	The beta oscillatory response to pulses of TMS applied over the M1 is higher in PD than in HS.
Fuggetta et al. (2005)	Alpha and beta	10 HS	Left M1	The decrease in alpha (8–12 Hz) activity at M1 was observed, with its amplitude synchronized to the rising TMS intensity.
Julkunen et al. (2013)	Alpha, beta and gamma	7 EPM1 6 HS	Left M1	The power and coherence of alpha (8–13 Hz), beta (13–30 Hz), and gamma (30–48 Hz) oscillations over M1 were reduced in patients with Unverricht-Lundborg disease.
Kirkovski et al. (2016)	Beta	22 ASD 20 HS	Right DLPFC Right M1 Right TPj	Increased levels of autistic traits were associated with decreased phase synchrony in the beta (13–30 Hz) band with TMS of M1.
Formaggio et al. (2016)	Alpha, beta, delta and theta	5 DoC 5 HS	Bilateral M1	The opposite pattern of TMS-induced EEG power in the alpha (8–12 Hz) and beta (13–30 Hz) bands at M1 was found in DoC compared to HS.
Casula et al. (2018)	Alpha and theta	16 HD 16 HS	Left M1 Left PM	The level of phase synchronization in response to M1 of TMS was found to be lower in individuals with Huntington's disease compared to healthy volunteers.
Borich et al. (2016)	Beta	10 chronic stroke 4 HS	M1	Significantly increased TMS-evoked beta (15–30 Hz frequency range) IPC was observed in the stroke group during ipsilesional M1 stimulation compared to controls during TCI assessment, but not at rest.
Pellicciari et al. (2018)	Alpha	13 stroke 10 HS	M1 PPC	The TMS-evoked alpha oscillations over the M1 were increased in stroke patients.
Groppa et al. (2013)	Alpha and beta (8–12 Hz, 13–30 Hz)	13 HS	Left M1	An enhancement of oscillatory interaction between the corresponding central regions of both hemispheres in the alpha band is induced by TMS.
Gordon et al. (2018)	Alpha and beta	12 HS	Left M1	TMS at 90% RMT resulted in a significant increase (50–200 ms) and a subsequent decrease (200–500 ms) in the power of alpha and beta oscillations. TMS at 110% RMT resulted in an additional increase in beta power in late latencies (650–800 ms).
Formaggio et al. (2023)	Beta	15 PD 10 HS	Left M1	Brain oscillations in PD transiently reset after TMS: beta power over M1 becomes comparable to that recorded in age-matched healthy subjects in the 2 s following TMS.
Tscherpel et al. (2020)	Alpha, beta and theta	28 stroke 15 HS	M1	A slowdown of TMS-induced prefrontal alpha was reported in stroke patients.
Ferrarelli et al. (2008)	Gamma (30–50 Hz)	16 SCZ 14 HS	Right M1	A decrease in TMS-evoked potentials in the gamma-band (30–50 Hz) and a reduced spreading of activation have been observed in SCZ compared to HS.
Ferrarelli et al. (2012)	Beta and gamma (30–50 Hz)	20 SCZ 20 HS	Prefrontal cortex Premotor cortex Motor cortex Parietal cortex	An almost 10 Hz decrease in TMS over the prefrontal cortex was observed in SCZ compared to HS.
Pigorini et al. (2011)	Alpha and gamma (8–13 Hz) (30–50 Hz)	5 HS 1 SCZ	Brodmann4 Brodmann6 Brodmann19	The first oscillations evoked by TMS over the occipital and frontal regions are in the gamma band, followed by the slower ones in the alpha band.
Bai et al. (2022)	Alpha and beta	33 acute stroke	DLPFC M1 SPL	A significantly lower natural frequency was observed in patients with post-stroke delirium compared to those without post-stroke delirium at the DLPFC.
Casula et al. (2022)	Gamma	60 AD 21 HS	Left DLPFC PC Left PPC	A significant reduction in frontal gamma activity was shown by AD patients compared to age-matched HS.
Canali et al. (2017)	Beta and gamma	18 BD 9 HS	Superior frontal gyrus (BA6)	The beta and gamma (20–50 Hz) oscillations induced by TMS in the prefrontal areas were found to decrease significantly in BD.
Pellicciari et al. (2017a)	Theta, alpha, beta and gamma	1 MDD	Bilateral DLPFC	A remarkable asymmetry of cortical oscillatory activity was revealed in the major depressive disorder, with prominent alpha-band (8–12 Hz) oscillations over the left DLPFC, whereas more fast frequencies (beta: 13–30 Hz and gamma: 30–50 Hz) were observed over the right DLPFC.
Vallesi et al. (2021)	Alpha, beta and theta	12 HS	Bilateral DLPFC	A higher beta frequency was revealed on the rDLPFC when stimulated by TMS compared with other brain regions.
Rosanova et al. (2009)	Alpha, beta and gamma	7 HS	Brodmann19 Brodmann7 Brodmann6	1. The main frequency of TMS-evoked EEG oscillations depends on the site of stimulation. 2. The natural frequency reflects the local properties of corticothalamic circuits. 3.The local natural frequency is preserved across a wide range of stimulation intensities.
Stanfield and Wiener (2019)	Alpha, beta and gamma	24 HS	Right Brodmann19 Brodmann7 Brodmann6	There is no linear relationship between the stimulus site and the natural frequency.

ASD, Autism Spectrum Disorder; BD, bipolar disorder; BPD, bipolar disorder; DLPFC, dorsolateral prefrontal cortex; DoC, disorders of consciousness; EPM1, Unverricht-Lundborg disease; EEG, electroencephalogram; HD, Huntington's disease; HS, healthy subjects; IPC, imaginary phase coherence; M1, primary motor cortex; MDD, major depression disorder; PM, premotor cortex; PPC, posterior parietal cortex; PC, precuneus; PD, Parkinson's disease; rDLPFC, right dorsolateral prefrontal cortex; SCZ, schizophrenia; SPL, superior parietal lobule; TPj, temporoparietal junction; TMS, transcranial magnetic stimulation; TCI, transcallosal inhibition.

Related studies examining single-pulse TMS induced/evoked brain oscillations are organized by site of stimulation. The initial knowledge of employing TMS-EEG to measure steady-state evoked/induced oscillations primarily came from the motor area. It was frequently used to explore the potential oscillatory mechanisms underlying brain diseases. TMS applied to M1 elicited beta/gamma band responses in healthy individuals, whereas a predominant frequency in a lower frequency range (11–27 Hz) was observed in schizophrenia (SCZ) patients (Canali et al., 2015). Common to these psychiatric conditions, a biological underpinning of slower gamma-band oscillations could be found in abnormal -aminobutyric acid (GABA) neurotransmission. TMS over M1 induced a brief period of synchronized activity in the beta (15–30 Hz) range in the vicinity (Paus et al., 2001). A higher beta (15–30 Hz) oscillatory response to TMS of M1 was found in Parkinson's disease (Van Der Werf et al., 2006). TMS at M1 showed a decrease in alpha (8–12 Hz), whose amplitude synchronized with the increase in TMS intensity (Fuggetta et al., 2005). TMS over M1 showed reduced power and coherence of alpha (8–13 Hz), beta (13–30 Hz), and gamma (30–48 Hz) oscillations in patients with Unverricht-Lundborg disease (Julkunen et al., 2013). Recent research suggests that increased levels of autistic traits are associated with decreased phase synchrony in the beta (13–30 Hz) band with TMS of M1 (Kirkovski et al., 2016). The opposite pattern of TMS-induced EEG power in the alpha (8–12 Hz) and beta (13–30 Hz) bands at M1 was found in patients with disorders of consciousness, compared to healthy subjects (Formaggio et al., 2016). Individuals afflicted with Huntington's disease exhibited a reduced level of phase synchronization in the theta and alpha bands (4–13 Hz) in response to TMS at M1, compared to healthy subjects (Casula et al., 2018). In stroke patients, an increase in TMS-induced imaginary phase coherence within the beta frequency range (15–30 Hz) was observed with TMS of the ipsilesional M1, compared to healthy subjects (Borich et al., 2016). An increase in TMS-evoked alpha (8–12 Hz) oscillatory activity at M1 was considered as a potential neurophysiological marker of stroke recovery (Pellicciari et al., 2018). In summary, single-pulse TMS applied to M1 can induce/evoke various oscillatory responses that are influenced by the neurological and psychiatric conditions of the individuals being studied. These responses provide insights into the oscillatory mechanisms underlying brain function and disease and may serve as biomarkers for certain conditions.

Apart from the motor cortex, TMS-induced oscillations in the DLPFC also attracted interest from researchers. A slowdown of TMS-induced prefrontal alpha was reported in stroke patients (Tscherpel et al., 2020). Significantly decreased TMS-induced frontal gamma (30–50 Hz) was frequently demonstrated in SCZ patients (Ferrarelli et al., 2008; Pigorini et al., 2011; Ferrarelli et al., 2012). A natural frequency analysis at the DLPFC demonstrated a significantly lower natural frequency in acute stroke patients who developed later post-stroke delirium compared to those who did not (Bai et al., 2023). Similar results were found in patients with Alzheimer's disease. Patients with Alzheimer's disease showed lower local gamma (30–50 Hz) activity compared to a healthy group when TMS was applied over the left DLPFC (Casula et al., 2022). Furthermore, it was revealed that there is a distinct decrease in natural frequencies, particularly in the beta and gamma (20–50 Hz) oscillations induced by TMS in the prefrontal areas in patients with schizophrenia and bipolar disorder (Ferrarelli et al., 2012; Canali et al., 2017). When TMS was applied over the bilateral DLPFC, a remarkable asymmetry of cortical oscillatory activity was revealed. There were prominent alpha-band (8–12 Hz) oscillations over the left DLPFC, while faster frequencies (beta: 13–30 Hz and gamma: 30–50 Hz) were observed over the right DLPFC in individuals with major depressive disorder (Pellicciari et al., 2017a).

A significant work investigated the main frequency of TMS-evoked EEG oscillations (natural frequency) in different brain regions. TMS consistently evoked dominant alpha-band (8–12 Hz) oscillations at the occipital cortex [Brodmann area (BA) 19], beta-band (13–20 Hz) oscillations at the parietal cortex (BA 7), and fast beta/gamma-band (21–50 Hz) oscillations at the frontal cortex (BA 6), which was referred to as a rostro-caudal gradient (Rosanova et al., 2009). However, later studies failed to replicate this rostro-caudal gradient (Stanfield and Wiener, 2019). It was demonstrated that both occipital and frontal lobe stimulation led to initial gamma band (30–50 Hz) oscillations, followed by slower oscillations in the TMS-evoked EEG oscillations (Pigorini et al., 2011). It has also been found that the phase reset and information flow induced by single-pulse TMS when stimulating the visual cortex occurs mainly in the theta frequency band, and that the intensity of TMS has a significant effect on the phase reset and propagation of theta oscillations (Kawasaki et al., 2014).

Measures of TMS-evoked activity, such as induced and evoked oscillations, can also serve to explore the potential for pharmacological interventions, monitor brain plasticity over time, and predict treatment outcomes (Yavari et al., 2016). A study of the influence of GABAergic activity on TMS-induced and evoked cortical oscillations revealed that early synchronization and late desynchronization of induced oscillations might be influenced by distinct inhibitory mechanisms. Specifically, early alpha-band synchronization showed an increase in the presence of GABAAR-mediated drive, facilitated by substances like zolpidem, diazepam, and alprazolam. Conversely, GABABR-mediated activity, enhanced by baclofen, led to a decrease in early alpha-band synchronization. Both GABAAR and GABABR activity were found to enhance late beta-band desynchronization. Furthermore, the GABABR agonist baclofen was associated with an increase in late alpha-band desynchronization. In short, early α-synchronization was increased by GABAAergic drugs and decreased by GABABergic drugs. The late α-desynchronization was increased by the GABABergic drug, while the late β-desynchronization was increased by both GABAAergic and GABABergic drugs. These results shed light on the intricate involvement of GABAergic activity in shaping induced and evoked cortical oscillations (Premoli et al., 2017). Subsequently, a study investigated the effects of anti-glutamatergic drugs (dextromethorphan, an NMDA receptor antagonist; perampanel, an AMPA receptor antagonist) and an L-type voltage-gated calcium channel blocker (nimodipine) on TMS-induced oscillations. The results indicated that the oscillations induced by the midline parietal area reflect glutamatergic signal propagation, which is mediated by AMPA receptor activation and occurs both within and between the cerebral hemispheres. Components at the mid-parietal lobe are likely involved in this long-range signal propagation (Belardinelli et al., 2021). These insights gained from pharmacological characterization of TMS-induced brain oscillations provide valuable information for understanding oscillatory abnormalities in neuropsychiatric disorders characterized by imbalanced excitation-inhibition processes.

## 4 Oscillatory entrainment by TMS

Entrainment refers to the phenomenon in which natural oscillations are driven by a periodic external force, causing the oscillating element to synchronize with the rhythmic external forces (Thut et al., 2011a). As a form of direct entrainment driven purely by external forces, it can be achieved through several non-invasive brain stimulation methods such as visual flicker, rTMS, transcranial direct current stimulation or transcranial alternating current stimulation. We will here focus on the oscillatory entrainment produced by rTMS (Table 3).

**Table 3:** Oscillation entrainment by TMS.

**References**	**TMS frequency**	**Measured brain oscillation**	**Targets**	**Main findings**
Thut et al. (2011b)	Individual alpha (9–11 Hz)	Individual alpha (9–11 Hz)	Parietal cortex	Local synchronization of parietal alpha activity occurred when targeting the underlying alpha generator with short bursts of alpha-rTMS (five pulses at the individual alpha frequency).
Hanslmayr et al. (2014)	Beta (18.7 Hz)	Beta (17.5–19.5 Hz)	Left inferior frontal gyrus	Entrainment of prefrontal beta oscillations was observed for a few cycles after the end of stimulation; memory was impaired only when synchronization occurred in the beta frequency range.
Romei et al. (2016)	Individual beta-frequency (14.7–22.6 Hz)	Individual beta-frequency (14.7–22.6 Hz)	Left M1HAND	TMS entrainment in the motor cortex is maximal at the individual beta frequency.
Albouy et al. (2017)	Theta (5 Hz)	Theta (4–6 Hz)	Left Intraparietal Sulcus	Rhythmic TMS entrained theta oscillations and improved the accuracy of working memory.

M1, primary motor cortex; M1HAND, hand representation of M1; rTMS, repetitive transcranial magnetic stimulation; TMS, transcranial magnetic stimulation.

**Figure 2:**
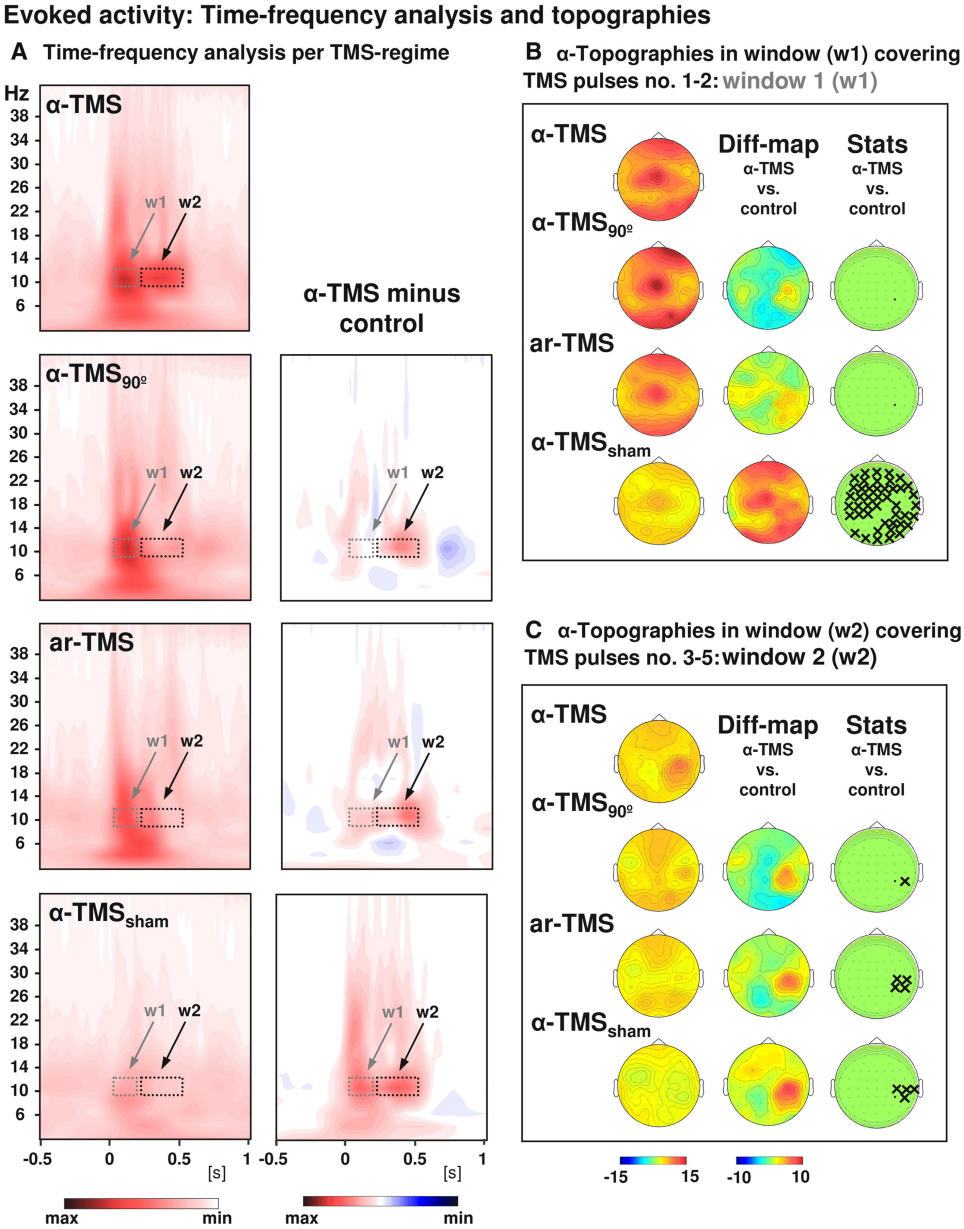
Grand-averaged time-frequency plots and topographical analysis in TMS entrainment. Comparison of alpha-TMS bursts (active alpha-TMS perpendicular to target gyrus) with all three control conditions, i.e., alpha-TMS_90_ (active alpha-TMS parallel to target gyrus), ar-TMS (active rapid-rate TMS in an arrhythmic regime perpendicular to target gyrus), and alpha-TMS_sham_ (inactive alpha-TMS). **(A)** Time-frequency plots for electrode CP4 (closest to TMS hot spot) for all conditions (left panels) and subtractions (alpha-TMS minus control, right panels). w1 and w2 indicate windows of distinct early and late effects (the windows cover the entire train, which lasted 400 ms). **(B)** Topographies of the TMS-evoked responses for alpha-layer activity in the early window (w1). **(C)** Topographies of the TMS-evoked responses for alpha-layer activity in the late window (w2). The columns represent grand-average maps (left column), difference maps (alpha-TMS minus controls; middle columns), and corresponding *t*-statistics (right columns). Xs indicate electrodes with statistically significant voltage differences in alpha-TMS relative to the corresponding control (with permission, from Thut et al., 2011b).

TMS-EEG studies have revealed an entrainment of brain oscillations during repetitive TMS. A study showed a local entrainment of parietal alpha oscillations with short bursts of alpha-rTMS at the right intraparietal sulcus (Figure 2) (Thut et al., 2011b). TMS of beta frequency on the left inferior frontal gyrus elicited entrainment of endogenous oscillations that outlasted the stimulation period by ~1.5 s (Hanslmayr et al., 2014). A recent study found that beta neural oscillations emerging from the sensorimotor area influence the modulation of motor response vigor (Uehara et al., 2023). Entrainment during short-burst rTMS of the beta rhythm applied to the motor cortex elicited responses that were strongest with TMS pulses at the individual's intrinsic beta peak frequency (Romei et al., 2016). In addition, endogenous theta oscillations were entrained by targeting rTMS to the left intraparietal sulcus, and such entrainment was relevant to an improvement in auditory working memory (Albouy et al., 2017). Based on these findings, a hypothesis was proposed that TMS-locked oscillations in the various frequency bands share a common neurophysiological origin with sustained spontaneous oscillations. Evidence in support of this hypothesis indicated that TMS-evoked alpha oscillations of the visual cortex react in a similar manner to top-down attentional modulation compared to endogenous oscillations. This strongly suggests that these TMS-evoked oscillations are produced by the same neuronal mechanisms as the targeted spontaneous oscillations (Herring et al., 2015). Moreover, it has been demonstrated that spontaneous oscillations have region- and network-specific effects, as manipulating oscillations in localized areas impacts other regions through a large-scale oscillatory network with corresponding frequency specificity (Okazaki et al., 2021). Building on these insights, a recent study has found that resting-state EEG biomarkers, particularly the spectral characteristics of alpha activity, can predict individual differences in the success of rhythmic-TMS entrainment and frequency modulation. These biomarkers act as a proxy for an individual's Arnold tongue (Trajkovic et al., 2024). This discovery not only provides a theoretical and experimental framework to explain the variability in outcomes across different rhythmic-TMS studies but also presents a potential biomarker and evaluative tool. These tools are crucial for developing the most optimal and personalized TMS protocols for both research and clinical applications.

## 5 Oscillation-dependent responses to TMS

Recent studies have revealed that ongoing cortical oscillations interact with brain responses to TMS. It has been demonstrated that the TMS-evoked cortical response is dynamically shaped by the intrinsic neural properties of the neurons at the time of stimulation, resulting in variable responses to external stimulation (Table 4).

**Table 4:** Oscillation-dependent responses to TMS.

**References**	**Frequency bands**	**Power**	**Phase**	**Assessment**	**Targets**	**Main findings**
Romei et al. (2008)	Alpha (8–14 Hz)	Low prestimulus power	/	Phosphenes	Occipital cortex	Low prestimulus alpha band power resulted in TMS inducing phosphenes, whereas high prestimulus alpha power failed to evoke a visual percept.
Sauseng et al. (2009)	Alpha (around 10 Hz)	Low prestimulus power	/	MEPs	M1	Low prestimulus alpha power resulted in TMS reliably inducing MEPs, while high prestimulus alpha power failed to induce MEPs.
Dugue et al. (2011)	Alpha (10 Hz)	/	Peak and next zero-crossing	Phosphenes	Occipital cortex	Cortical activation occurring between the peak and the next zero-crossing of the occipital EEG alpha oscillation phase is most likely to produce phosphenes.
Bergmann et al. (2012)	Slow oscillation (1 Hz)	/	Up-states	MEPs\TEPs	M1HAND	The MEP amplitude was small and delayed during sleep compared to wakefulness, whereas sleep TEPs were similar to spontaneous slow oscillations. Both MEPs and TEPs were consistently larger when evoked during slow oscillation up-states than during down-states.
Zrenner et al. (2018)	Mu (8–12 Hz)	/	Negative peak	MEPs	M1	Repeatedly stimulating at the negative peak (high-excitability state) of the mu rhythm with 100-Hz TMS triplets resulted in a long-term potentiation-like effect, while no change occurred if the same rTMS was triggered at the positive peak (low-excitability state) or at a random mu-rhythm phase.
Thies et al. (2018)	Mu (8–14 Hz)	High prestimulus power	/	MEPs	M1	There was a weak positive relationship between spontaneous sensorimotor mu-power fluctuations at rest and MEP amplitude.
Desideri et al. (2018)	Mu (8–12 Hz)	/	Negative peak	MEPs	M1 ipsi-and contralateral	Mu-rhythm-phase-dependent burst-rTMS did not significantly change any TMS-EEG measures of cortical excitability, although there was a significant differential and reproducible effects on MEP amplitude.
Stefanou et al. (2018)	Mu (8–12 Hz)	/	Negative peak	Short-interval interhemispheric inhibition (SIHI)	Left and right M1	The strongest short-interval interhemispheric inhibition was found when the two M1 were in phase for the high-excitability state (trough of the mu-rhythm), whereas the weakest short-interval interhemispheric inhibition occurred when they were out of phase and the left M1 was in a low-excitability state (peak of the mu-rhythm).
Zrenner B. et al. (2020)	Alpha (8–12 Hz)	/	Negative peak	Alpha activity	DLPFC	RTMS triggered at the trough of instantaneous alpha oscillations (alpha-synchronized rTMS) reduced resting-state alpha activity in the left DLPFC and increased TMS-induced beta oscillations over frontocentral channels in patients with major depressive disorder.
Schaworonkow et al. (2018b)	Mu (8–12 Hz)	/	Negative peak	MEPs	Left M1	The amplitude of MEP was modulated by mu-phase across a wide range of stimulation intensities, with larger MEPs when TMS was applied at the trough of the mu-rhythm. The largest relative MEP modulation was observed for weak intensities, while the largest absolute differences were observed for intermediate intensities.
Bergmann et al. (2019)	Mu-alpha (8–14 Hz)	High power	Troughs and rising flanks	MEPs	Left M1	MEP amplitudes were facilitated during high power troughs and rising flanks of the mu-oscillation, but they were not altered during peaks and falling flanks. No modulation of short-latency intracortical inhibition was observed.
Desideri et al. (2019)	Mu (8–12 Hz)	/	Negative peak	TEPs	M1	High-intensity TMS applied at the trough relative to positive peaks of the mu-rhythm was associated with higher absolute amplitudes of TEPs at 70 ms (P70) and 100 ms (N100), while low-intensity TMS applied at the trough of the mu-rhythm enhanced the N100.
Ogata et al. (2019)	Alpha and beta (10–15 Hz)	High alpha power and low beta power	/	MEPs	Left M1	The prestimulus higher-power alpha or low-beta bands produced larger MEPs only in the high-intensity eye open condition.
Baur et al. (2020)	Mu (9–13 Hz)	/	Negative\ positive\ random peak	LTP\LTD	Left M1	RTMS at the trough of the mu-rhythm showed a trend toward long-term potentiation-like corticospinal plasticity. RTMS at the positive peak of the mu-rhythm induced a significant long-term depression-like corticospinal plasticity. RTMS at random phase resulted in a trend toward long-term depression-like plasticity.
Wischnewski et al. (2022)	Mu (8–13 Hz) and beta (14–30 Hz)	Mu power, but not beta power	Trough and rising phase	MEPs	M1HAND	MEPs were larger at the mu trough and rising phase, and smaller at the peak and falling phase. MEPs were larger at the beta peak and falling phase, and smaller at the trough and rising phase. Mu power, but not beta power, was positively correlated with corticospinal output.
Gordon et al. (2022)	Theta (4–7 Hz)	/	Negative peak	Amplitude and power of the EEG responses \working memory performance	Left dorsomedial prefrontal cortex(DMPFC)	Compared to the same stimulation at the positive peak or random phase, rTMS performed at the trough of the prefrontal theta oscillation increased the amplitude and power of the EEG responses, and improved performance in a working memory task.
Bai et al. (2022)	Alpha (7–13 Hz) and theta	Both high alpha-power states	/	TEPs	Left M1HAND	Both high alpha-power states (stimulated - non-stimulated hemisphere: alpha-theta/alpha-alpha state) in the stimulated left sensorimotor cortex increased the P25 amplitude. The N45 peak was significantly increased in the alpha-alpha state. The P70 peak was significantly increased in the alpha-theta state.
Zrenner et al. (2022)	Mu (8–13 Hz)	/	Negative peak	MEPs	Postcentral gyrus, somatosensory cortex/precentral gyrus, premotor cortex	The trough vs. positive peak of the sensorimotor mu-rhythm, as extracted from the postcentral gyrus, correlated with states of high vs. low corticospinal excitability. No significant correlation was found for the sensorimotor mu-rhythm extracted from the precentral gyrus.

DLPFC, dorsolateral prefrontal cortex; DMPFC, dorsomedial prefrontal cortex; EEG, electroencephalogram; LTP, long-term potentiation-like; LTD, long-term depression-like; MEPs, motor evoked potentials; M1, primary motor cortex; M1HAND, hand representation of M1; rTMS, repetitive transcranial magnetic stimulation; SIHI, Short-interval interhemispheric inhibition; TEPs, TMS evoked potentials; TMS, transcranial magnetic stimulation.

The first direct evidence came from TMS at the occipital cortex. It has been illustrated that reports of phosphenes depend on the alpha power of ongoing oscillations immediately preceding occipital TMS. Specifically, low alpha power increases the likelihood of perceiving phosphenes, while high alpha power does not (Romei et al., 2008). Similarly, in the motor cortex, the amplitude of motor evoked potentials (MEPs) was negatively correlated with the alpha power of the primary motor cortex before the TMS pulses, when testing with TMS intensities close to motor threshold (Thies et al., 2018). However, when testing with clearly suprathreshold TMS intensities, a positive relation was consistently found, with high alpha- (or mu-) power in motor cortex associated with larger MEP amplitudes (Ogata et al., 2019).

In addition to oscillation power, studies have shown that the oscillatory phase plays a crucial role in TMS-evoked/induced responses. For instance, phosphene reports varied with the instantaneous phase of the alpha oscillation in the occipital cortex prior to the TMS pulse. Specifically, the EEG response was highly phase-locked with the 400-ms pre-TMS alpha oscillations when phosphene reports were reported, while there was a large phase variation in the case of no phosphene reports (Dugue et al., 2011). In the motor cortex, cortical excitability is dependent on the mu-oscillatory phase. MEPs are larger at the mu-trough and rising phase and smaller at the peak and falling phase (Zrenner B. et al., 2020). In the case of beta, MEPs are smaller at the oscillation trough and rising phase, and larger at the peak and falling phase (Wischnewski et al., 2022), but this observation was not as of yet replicated, and it was not ruled out that the observations on beta were simply and non-specifically caused by harmonics of the mu-oscillation in the beta frequency range. A subsequent study located the anatomical origin of the mu-rhythm in this relationship, revealing that the phase of the mu-rhythm from the somatosensory cortex, rather than the motor cortex, correlates with corticospinal excitability. Specifically, when TMS was triggered at the negative peak of the EEG mu-rhythm, it elicited larger MEPs, indicating a heightened state of corticospinal neuron excitability. This finding suggests that the mu-rhythm phase from the somatosensory cortex is a better predictor of corticospinal excitability than that from the motor cortex, highlighting the involvement of specific neural pathways from the somatosensory cortex to the primary motor cortex, even in simple cases (Zrenner et al., 2022).

The dependency of cortical responses to TMS on the mu-phase could also be reflected by the amplitude of the TEPs and by the power of the TMS-induced EEG oscillations (Desideri et al., 2019). During non-REM slow oscillation sleep, TMS evoked larger MEPs and TEPs during slow oscillation EEG up-states, whereas smaller MEPs and TEPs were observed during the down-states (Bergmann et al., 2012).

Moreover, the phase of brain oscillations significantly influences the effects of TMS on cortico-cortical connectivity, with TMS pulses during the trough of mu-rhythms enhancing interhemispheric synchronization (Momi et al., 2022). The oscillatory phase not only affects local cortical excitability but also TMS responses in cortico-cortical networks. The communication through coherence theory suggests that effective cortico-cortical connectivity depends on the synchronization of the instantaneous phase of neuronal oscillations of nodes in a network (Fries, 2005). Short-interval interhemispheric inhibition, a marker of interhemispheric effective connectivity, was maximally expressed when paired-pulse stimulation was triggered while the phases of the mu-rhythms in the motor cortices of the two hemispheres were in synchrony (Stefanou et al., 2018). Such bihemispheric sensorimotor oscillatory network states also showed a similar effect on TEPs. The P25 TEP component of the stimulated sensorimotor cortex distinctly increased when the stimulated cortex is dominated by alpha rhythm. Bilateral N45 is significantly inversely correlated with alpha power in a bilateral alpha-alpha state. P70 at the stimulated sensorimotor cortex shows a positive correlation with the theta power in an alpha-theta state (Bai et al., 2022). This underscores the crucial role of alpha-band cortico-cortical phase synchronization in effective connectivity within the motor network, suggesting a key role for alpha-band synchronization in motor control and coordination (Zazio et al., 2021).

**Figure 3:**
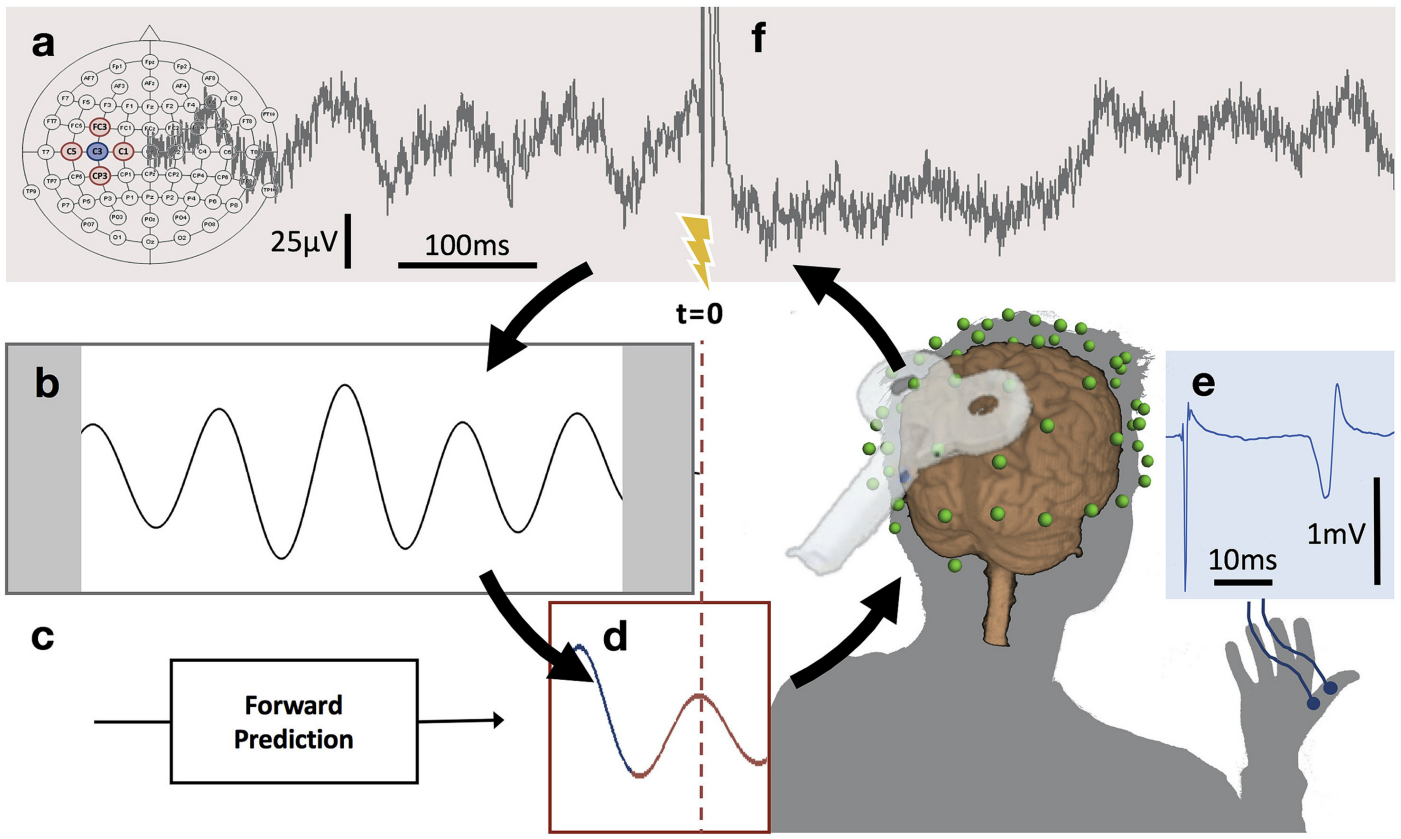
μ-oscillation phase-triggered brain stimulation apparatus. **(A)** Scalp EEG raw data derived from a 5-point sum-of-difference operation centered on the C3 EEG electrode (Hjorth-C3) over left sensorimotor cortex is streamed to a real-time system with 3 ms latency where the processing algorithm is computed at a rate of 500 Hz. **(B)** A 500 ms sliding window of data is 8–12 Hz bandpass filtered forward and backward and edge artifacts (shaded) are removed. **(C)** Coefficients for an autoregressive model are calculated from the filtered data. **(D)** The signal is forward predicted (red trace), phase is estimated at time zero (*t* = 0) using a Hilbert transform and the TMS stimulator is triggered when a pre-set phase condition is met. **(E)** TMS of the hand area of left primary motor cortex produces a MEP in right hand muscles recorded with surface EMG. **(F)** Recovery of the μ-oscillation ~300 ms after the TMS pulse (for interpretation of the references to color in this figure legend, the reader is referred to the web version of this article) (with permission, from Zrenner et al., 2018).

The relationship between spontaneous oscillations and brain responses to TMS directly leads to the hypothesis that brain state-dependent stimulation, in which rTMS triggered by instantaneous phase, power or synchronization of oscillations, will be able to enhance its neuro-modulation effects. With the advent of this strategy, it has become possible to excite or inhibit target neurons with the same stimulation protocol, but with TMS pulses locked with different oscillation phases (Zrenner et al., 2016). Based on the combination of EEG oscillations and TMS (i.e., EEG-TMS), identical rTMS on the through (high-excitability) vs. positive peak (low-excitability) of the sensorimotor mu-rhythm, led to long-term potentiation-like vs. no change in corticospinal excitability (Figure 3) (Zrenner et al., 2018). Similarly, 1-Hz rTMS led to a trend of long-term depression (LTD)-like plasticity at random phases, and significant LTD-like plasticity with rTMS at the positive peak condition (i.e., the low-excitability state) (Baur et al., 2020). In a non-motor region, a phase-dependent rTMS of theta rhythms in the left dorsomedial prefrontal cortex was performed using real-time EEG-TMS. This work revealed that rTMS at the trough increased the single-pulse TMS-induced prefrontal theta power and theta-gamma phase-amplitude coupling, and decreased the response time of working memory tasks (Gordon et al., 2021, 2022). Subsequently, an application of EEG-TMS in patients with major depressive disorder revealed that specifically synchronizing rTMS pulses to the trough of instantaneous alpha oscillations in the DLPFC reduced resting-state EEG alpha power, a marker of depression severity (Zrenner B. et al., 2020). It supports evidence that EEG-TMS has significant potential to be a feasible and safe neuromodulation strategy for patients with brain network and excitability disorders (Zrenner and Ziemann, 2024).

## 6 Discussion

In well-defined neural networks, spontaneous neural activity fluctuates dynamically over time, suggesting more than a simple causal relationship between cortical oscillatory activity and cortical excitability modulation (Goldman et al., 2002; Damoiseaux et al., 2006). Therefore, this review explores the complex interactions between TMS and brain oscillations, focusing on three aspects: the effects of TMS on oscillations, the effects of oscillations on TMS, and the interactions between the two.

Firstly, regarding the effects of TMS on brain oscillations, rTMS and single-pulse TMS each demonstrate distinct mechanisms in modulating intrinsic brain oscillations. Through exogenous stimulation at various frequencies, rTMS can selectively modulate neural oscillations within specific brain regions, while single-pulse TMS is capable of inducing brain oscillations, thereby revealing the cortex's natural frequency. The following discussion provides a detailed examination of these effects. Brain oscillations reflect the synchronization of the activity of large neuronal populations, rhythmically switching between states of low and high excitability (Schroeder and Lakatos, 2009), which is achieved through the interactions and connections between neurons. The power of spontaneous brain oscillations fluctuates with processing of external stimuli (Hanslmayr et al., 2007; van Dijk et al., 2008), and stimuli with specific frequencies can affect the synchronization of neural activities, thereby modulating brain oscillations (Schutter and Hortensius, 2011). Low-frequency rTMS, in the 1 Hz range, can decrease the excitability of the motor cortex, whereas high-frequency rTMS in the range of 10–20 Hz appears to temporarily increase cortical excitability (Kobayashi and Pascual-Leone, 2003). This modulation alters neuronal connections and activity, subsequently leading to corresponding adjustments in the brain's oscillatory patterns and functions. Specifically, high-frequency (beta, gamma) power is often correlated with cognitive functioning and levels of consciousness, while low-frequency (delta, theta) power is associated with sleep, memory, and the processing of sensory functions such as vision and hearing (von Stein and Sarnthein, 2000; Başar et al., 2001). Previous research has shown that impaired cognitive function is linked to decreased high-frequency power in patients with Alzheimer's disease (Liu et al., 2022), disorders of consciousness (Xia et al., 2017), or chronic non-fluent aphasia (Dammekens et al., 2014). Applying a specific frequency rTMS protocol, such as 10 or 20 Hz, to these patients can increase the high-frequency power and thereby enhance cognitive function. This frequency-specific modulation of oscillatory patterns by TMS holds therapeutic potential for neurological and psychiatric disorders. Notably, rTMS effects are cumulative and result from multiple sessions, rather than isolated single-session effects. This distinction aligns with findings indicating that the cumulative impact of repeated stimulation enhances therapeutic and neurophysiological outcomes, potentially through mechanisms of neural plasticity and entrainment across sessions. Furthermore, the variability in rTMS effects may arise due to factors such as individual differences, patient-specific pathological states, and variability in stimulation parameters (e.g., frequency, intensity, and target location). These factors can significantly influence the efficacy and consistency of rTMS outcomes, as shown in recent studies on rTMS and brain oscillations.

Moreover, the use of controlled perturbations introduced through single-pulse TMS allows for the identification of local frequency characteristics in specific cortico-thalamic regions and enables observation of their interactions at the whole-brain level (Rosanova et al., 2009). Electrical activity in the resting brain results from complex thalamocortical and corticocortical interactions and oscillations occur in a localized regional manner. When TMS targets different areas, it produces a complex EEG response characterized by strong fluctuations at the natural frequency of the stimulated region and weaker fluctuations near the natural frequency of distant regions (Paus et al., 2001; Massimini et al., 2005). However, electrical activity decays during conduction, and each cortical region tends to maintain its own natural frequency, indicating that the observed oscillations reflect primarily local physiological mechanisms. Additionally, detecting natural frequencies through TMS-EEG holds diagnostic potential and clinical value. Altered membrane properties of cortical and subcortical (especially thalamic) neuronal subpopulations, as well as changes in their connectivity patterns, underlie various neurological and psychiatric disorders (Hughes and Crunelli, 2005; Llinás et al., 2007). These changes may result in significant and noticeable changes in their oscillatory properties. These alterations may be diffuse or localized, making it potentially crucial to map the natural frequencies of different cortical regions in various neuropsychiatric disorders, such as depression, epilepsy and disorders of consciousness. In summary, the effects of TMS on brain oscillations underscore its value as an effective tool for modulating and investigating brain oscillations, enhancing our understanding of frequency-specific cortical functions and offering potential therapeutic avenues for disorders characterized by oscillatory imbalances.

The approach of brain state-dependent TMS on brain oscillations provides valuable insights into how brain oscillations affect the outcomes of TMS by leveraging real-time information about the transient state of the brain through endogenous rhythmic phase-specific rTMS to induce long-term changes in excitability or connectivity within the stimulated network (Figure 3) (Zrenner et al., 2018). This real-time feedback mechanism helps us better understand brain changes in different states (e.g., awake, asleep, focused, or pathological).

To date, research data has shown that the power of alpha oscillations in the occipital lobe is inversely correlated with TMS-induced visual cortical excitability, whereas the power of the alpha (mu) rhythm in the sensorimotor cortex is positively correlated with sensorimotor cortical excitability. The findings in occipital cortex support the pulsed inhibition hypothesis (Klimesch et al., 2007), suggesting that a decrease in alpha activity reflects a state of enhanced cortical excitability, while an increase in alpha activity leads to a state of cortical idling (Pfurtscheller, 2001), or even active inhibition (Foxe et al., 1998). This spontaneous rhythmic oscillation associated with phosphene perception is location-specific. However, findings in sensorimotor cortex do not align with this common view, which may be due to the more complex relationship between the power of the mu-rhythm and excitability in somatosensory (Nikouline et al., 2000; Linkenkaer-Hansen et al., 2004; Jones et al., 2010; Zhang and Ding, 2010; Ai and Ro, 2014) and motor cortex (Zarkowski et al., 2006; Sauseng et al., 2009).

The mu-phase-based EEG-TMS studies summarized in Table 4 preselected subjects with strong pericentral mu-activity, while corticospinal excitability was not significantly modulated by the phase of the mu-rhythm in non-preselected participants (Madsen et al., 2019; Karabanov et al., 2021). Therefore, future studies need to elaborate further on the specific conditions and populations, in which the human sensorimotor cortex responds to mu-rhythm-dependent TMS. In addition to mu-rhythm phase-dependent EEG-TMS studies of sensorimotor cortex, real-time EEG-TMS showed phase-specific effects of the ongoing theta-rhythm in dorsomedial prefrontal cortex (Gordon et al., 2022), suggesting the potential for a generalizable operation of EEG-TMS in human cortex. For specific neural oscillations associated with psychiatric and neurological disorders, EEG-TMS treatment can target these abnormal activities, improving both precision and effectiveness. We therefore anticipate that therapeutic brain state-dependent stimulation will become a major strategy for effectively treating various neurological and psychiatric disorders in the future.

From this, we can observe not only that TMS has an excitatory modulating effect on the cerebral cortex (Schecklmann et al., 2015; Xia et al., 2017), but that stimulus intensity, inter-stimulus interval, and pulse configuration of TMS have the potential to actively alter the intrinsic cortical state. Moreover, the phase and power of ongoing cortical oscillations, as well as the synchronization of oscillatory activity between areas within one hemisphere or even between the hemispheres, significantly impact the excitability state of the stimulated cortex. This implies a complex interaction between TMS and the ongoing neuronal activity intrinsic to the cortex (Madsen et al., 2019). Additionally, based on the results of TMS studies with single pulses, different individual conditions exhibit region-specific oscillation frequencies (Canali et al., 2015; Okazaki et al., 2021), which guided us to directly manipulate brain dynamics with rTMS close to these specific frequencies (natural frequencies). The entrainment approach overcomes the limitation of using fixed stimulation frequency parameters for modulating brain oscillations by employing periodic pulses that are close to the local spontaneous oscillation frequency, which facilitates the rhythmic synchronization of neurons (Thut et al., 2011a) (Figure 2). This rhythmic external input enhances the phase coupling between endogenous oscillations and external stimuli, effectively modulating brain oscillations and influencing other regions through a large-scale oscillatory network. In brief, the phenomenon of entrainment provides an important perspective for understanding the interaction between TMS and brain oscillations. It not only demonstrates its potential in researching fundamental neural mechanisms but also offers new insights for clinical applications, particularly in improving cognitive functions and treating neurological disorders. Future research can further explore how to optimize parameters to maximize the benefits of this oscillatory modulation capability, thereby achieving more effective treatment strategies.

Methodologically, We generally study TMS-induced/evoked oscillations using time-frequency representation (TFR) approaches. Essentially, TFR involves the spectral decomposition of the EEG signal, resulting in a matrix that represents oscillatory power as a function of both time and frequency (employing methods such as wavelet transforms, Hilbert transform, and short-term Fourier analysis). There are three definitions for TMS-induced/evoked oscillations based on the methods used to extract cortical oscillations from EEG signals triggered by TMS: evoked oscillatory response, induced oscillatory response, and total oscillation response (Roach and Mathalon, 2008; Herrmann et al., 2014). Moreover, it is crucial to differentiate among these various approaches to accurately capture neural oscillatory responses, as specific interactions between TMS and brain states can generate intricate cortical patterns. The total oscillation response comprises both the evoked oscillatory response and the induced oscillatory response. The former is time- and phase-locked to the TMS pulse, while the latter is only phase-locked to the TMS pulse. Despite a significant amount of research on neural oscillations mentioning the induced oscillatory response, many studies fail to clarify that this response is obtained by subtracting the evoked oscillatory response from the total oscillation response. Hence, it is essential to determine the most suitable approach for extracting cortical oscillations from TEPs according to the research hypothesis and provide a detailed description of it (Pellicciari et al., 2017b). In addition, the accuracy of real-time phase detection algorithms depends heavily on the signal-to-noise ratio (Zrenner C. et al., 2020). Algorithmic improvements [e.g., individually tailored spatial filters, such as computed by spatial-spectral decomposition (SSD), and an individualized classifier of cortical excitability states] make it possible to accurately identify the cortical excitability state and extract the optimal oscillatory EEG signals. This enables to enhance the effectiveness of oscillatory state-dependent individualized therapeutic brain stimulation (Schaworonkow et al., 2018a; Metsomaa et al., 2021).

## 7 Conclusion

In conclusion, conventional fixed open-loop rTMS protocols do not take into account continuous variations in brain state, a capability needed for individualized and precise modulation of brain activity. One relatively simple approach to improve traditional rTMS is entrainment by applying rhythmic stimulation at or close to the brain's natural oscillations. This approach promotes phase coupling between endogenous oscillations and external stimulation. Going one leap ahead, brain state-dependent real-time EEG-TMS enables informed decisions on when to stimulate the brain, based on the phase or power of ongoing oscillations, but potentially also more complex spatio-temporal EEG signatures to guide individualized and precise neuromodulation. It is expected that therapeutic brain state-dependent stimulation will become a major strategy for the effective treatment of a broad variety of neurological and psychiatric disorders.
